# Synergy between CD40 and MyD88 Does Not Influence Host Survival to *Salmonella* Infection

**DOI:** 10.3389/fimmu.2015.00460

**Published:** 2015-09-14

**Authors:** Ulf Alexander Wenzel, Maria Fernandez-Santoscoy, Miguel A. Tam, Pia Tegtmeyer, Mary Jo Wick

**Affiliations:** ^1^Department of Microbiology and Immunology, Mucosal Immunobiology and Vaccine Center (MIVAC), Institute of Biomedicine at Sahlgrenska Academy, University of Gothenburg, Gothenburg, Sweden; ^2^BioLegend, San Diego, CA, USA; ^3^TWINCORE Centre for Experimental and Clinical Infection Research, Institute for Experimental Infection Research, Hannover, Germany

**Keywords:** dendritic cell, *Salmonella*, NFκB, MyD88, CD40

## Abstract

Previous studies using purified toll-like receptor (TLR) ligands plus agonistic anti-CD40 antibodies showed that TLRs and CD40 can act synergistically on dendritic cells (DCs) to optimize T cell activation and Th1 differentiation. However, a synergistic effect of TLRs and CD40 during bacterial infection is not known. Here, we show that mice lacking the TLR adaptor MyD88 alone, or lacking both MyD88 and CD40 [double knockout (DKO) mice], are compromised in survival to *Salmonella* infection but have intact recruitment of neutrophils and inflammatory monocytes as well as unaltered abundance of DC subsets and DC activation in infected tissues. In contrast to infected wildtype and CD40^−/−^ mice, both MyD88^−/−^ mice and DKO mice lack detectable serum IFN-γ and have elevated IL-10. A synergistic effect of TLRs and CD40 was revealed in co-culture experiments where OT-II T cell proliferation was compromised when DKO DCs were pulsed with OVA protein and OVA_323–339_ peptide, but not with heat-killed *Salmonella* expressing OVA (HKS_OVA_), relative to MyD88^−/−^ DCs. By contrast, MyD88^−/−^ or DKO DCs pulsed with any of the antigens had a similar ability to induce IFN-γ that was lower than WT or CD40^−/−^ DCs. DKO DCs pulsed with HKS_OVA_, but not with OVA or OVA_323–339_, had increased IL-10 relative to MyD88^−/−^ DCs. Finally, HKS_OVA_-pulsed MyD88^−/−^ and DKO DCs had similar and low induction of NFκB-dependent and -independent genes upon co-culture with OT-II cells. Overall, our data revealed that synergistic effects of CD40 and MyD88 do not influence host survival to *Salmonella* infection or serum levels of IFN-γ or IL-10. However, synergistic effects of MyD88 and CD40 may be apparent on some (IL-10 production) but not all (OT-II proliferation and IFN-γ production) DC functions and depend on the complexity of the antigen. Indeed, synergistic effects observed using purified ligands and well-defined antigens may not necessarily apply when complex antigens, such as live bacteria, challenge the immune system.

## Introduction

Dendritic cells (DCs) are sentinels of the host, spread throughout the tissues to scan for antigens. Antigen encounter in peripheral tissues results in processing and DC migration to draining lymph nodes for antigen presentation to naïve T cells. To recognize infections and initiate an innate immune response, which ultimately culminates in adaptive immunity, DCs are equipped with a battery of pattern recognition receptors of which Toll-like receptors (TLRs) are one of the most important families. TLRs recognize an array of microbial structures, and ligation of most TLRs results in association with the adaptor protein MyD88 (1, 2). This leads to the recruitment of members of the IKK complex that degrade IκB resulting in activation of the so-called canonical NFκB pathway (1–4). This alters gene expression, induces cytokine production, and influences the maturation status of the DC by upregulating co-stimulatory molecules, such as CD80, CD86, and CD40, among other effects (1, 5). TLR4, which recognizes LPS, can also initiate signaling through a MyD88-independent pathway that utilizes the alternate adaptors Toll/IL-1R domain-containing adaptor-inducing IFN-β (TRIF) and translocation-associated membrane protein (TRAM), activating the non-canonical NFκB pathway (1–4).

The crosstalk between antigen-presenting DCs and antigen-specific T cells results not only in activation of the T cell but also in reciprocal DC activation. The later is characterized by upregulation of co-stimulatory molecules as well as secretion of cytokines (6). It has been demonstrated that the duration of the DC–T cell interaction influences the ability of the T cell to proliferate, survive, and differentiate to fully activated effector cells (7, 8). Furthermore, weak and transient stimulation of DCs induces their migration to the draining lymph node while strong and persistent signaling is required to induce DC cytokine secretion (9), which, in turn, directs the subsequent T cell response.

In addition to peptide/MHC-TCR binding, recognition of members of the TNF (tumor necrosis factor) receptor family on DCs and the corresponding ligand on T cells influences the ensuing DC–T cell interaction (10). One of the best characterized members of this family is CD40, which binds CD40L on activated T cells. This interaction triggers signaling in DCs via the NFκB-inducing kinase (NIK), resulting in activation of the non-canonical NFκB2 pathway (11, 12). Moreover, using purified TLR ligands or antigen plus agonistic anti-CD40 antibody, it was shown that the canonical NFκB pathway and non-canonical pathways act synergistically in DCs, optimizing T cell activation and Th1 differentiation (12–16). Additionally, it was demonstrated that the level of CD40 expression on DCs influences differentiation of regulatory T cells (Treg) (17). Moreover, DCs need CD40 stimulation to cross-present antigens and initiate cytotoxic activity in CD8 T cells (12, 18, 19).

Studies investigating synergistic signaling in DCs that influences the T cell response have been performed using purified TLR ligands or other defined structures (i.e., α-galactoceramide), agonistic anti-CD40 antibodies, and protein antigen (15, 16, 20, 21). However, the influence of synergistic signals delivered into DCs during bacterial infection, where TLR activation results from multiple ligands expressed naturally by bacteria, on the immune response to the bacteria is not understood. We thus investigated the synergistic effect of MyD88-dependent NFκB signaling and CD40-dependent NFκB2 signaling in the T cell response against the pathogen *Salmonella enterica* serovar Typhimurium.

## Materials and Methods

### Mice

C57BL/6 mice (called WT mice) were purchased from Charles River (Sulzfeld, Germany). MyD88^−/−^ mice and OT-II mice were generously provided by S. Akira and S. Schoenberger, respectively. All mice were more than 10 generations on a C57BL/6 background and the genotypes were screened by PCR. Ly5.1-expressing OT-II mice were obtained by breeding Ly5.1^+^ C57BL/6 mice with (Ly5.1^−^) OT-II mice. MyD88^−/−^CD40^−/−^ double knockout (DKO) mice were generated by crossing MyD88^−/−^ and CD40^−/−^ mice and intercrossing F1 offspring (MyD88^+/−^CD40^+/−^). Generating DKO pups from MyD88^+/−^CD40^+/−^ × MyD88^+/−^CD40^+/−^ breeding required antibiotic treatment Hippotrim vet (Bayer Animal Health, Copenhagen, Denmark) in the drinking water of breeding cages. Hippotrim was administered after initial observations that survival of pups was poor and Hippotrim improved offspring survival. Following genotyping of offspring, all further breeding was performed using MyD88^+/−^CD40^−/−^ females and MyD88^−/−^CD40^−/−^ males with Hippotrim treatment and the predicted Mendelian distribution of mouse strains was obtained. Weaned offspring survived well and did not require Hippotrim. Mice used as CD40-deficient throughout the study were littermates to DKO mice (from the MyD88^+/−^CD40^−/−^ × MyD88^−/−^CD40^−/−^ breeding) whose genotype was MyD88^+/−^CD40^−/−^ and are referred to as CD40^−/−^ mice. Mice were bred at the Laboratory for Experimental Biomedicine animal facility of the University of Gothenburg, used between 8 and 12 weeks of age and provided food and water *ad libitum*. All experiments were performed following protocols approved by the regional animal ethics committee (Permit # 212/2013) and institutional animal use and care guidelines were followed.

### Bacterial strains and infection of mice

*Salmonella enterica* serovar Typhimurium (called *S. typhimurium*) χ4550 is an SR11 derivative of reduced virulence (22). *S. typhimurium* χ4550-OVA (called *Salmonella*-OVA) expresses the model antigen ovalbumin (OVA) in a balanced-lethal vector system to maintain OVA expression *in vivo* in the absence of antibiotic selection and was cultured as reported previously (23). The concentration of bacteria was estimated by OD_600_. Mice were given 0.1 mL of 1% NaHCO_3_ intragastrically and after 10 min, 2 × 10^8^ bacteria were given intragastrically in 0.1 mL of PBS. The bacterial dose administered, as well as the bacterial load in organs at the time of sacrifice, was determined after plating serial dilutions of the bacterial suspension on Luria–Bertani plates.

### Cell preparation

Single-cell suspensions from spleen, mesenteric lymph nodes (MLN), Peyer’s patches (PP), and small intestine lamina propria (siLP) were prepared as described previously (23). The total number of cells per organ was determined by trypan blue exclusion. For Figure 4, CD11c-expressing cells were enriched from single-cell suspensions from spleen pooled from three mice per group per experiment using N418 magnetic beads (Miltenyi Biotec, Bergisch Gladbach, Germany) and AutoMACS (Miltenyi Biotec). CD4^+^ T cells from naive OT-II mice were isolated using the CD4^+^ T cell isolation kit from Miltenyi following the manufacturer’s protocol. Cells were >85% pure.

### Serum preparation for cytokine detection

Serum was collected after clotting of blood from the tail vein for 2 h at RT followed by 10 min centrifugation at 13,000 × *g*. Serum was collected and stored in aliquots at −80°C until further analysis.

### Flow cytometry

Before staining, cells were incubated on ice for at least 10 min with the 2.4G2 antibody to block Fc receptors. Multiparameter flow cytometry was performed using a LSRII flow cytometer with Diva software (BD Biosciences, San Diego, CA, USA). Analysis was performed using FlowJo software (Tree Star Inc., Ashland, OR, USA). Cell viability was evaluated by staining cells with either 7-aminoactinomycin D (7AAD, Sigma-Aldrich) or Live/Dead Fixable Aqua Dead Cell Stain (Life Technologies) according to the manufacturer’s recommendation. Antibodies used were as follows: anti-CD11c (HL3 or N418), anti-CD8α (53-6.7), anti-MHCII (M5/114), anti-CD11b (M1/70), anti-CD103 (M290), anti-Ly6C (AL21), anti-Ly6G (1A8), F4/80 (CI:A3-1), B220 (RA3-6B2), anti-CD4 (L3T4), NK1.1 (PK136), anti-CD80 (16-10A1), and anti-CD86 (GL1). Antibodies were purchased from BD Biosciences (BD Pharmingen, San Diego, CA, USA), from BioLegend (San Diego, CA, USA) or eBioscience (San Diego, CA, USA). Gates were set using fluorescent minus one controls or isotype-matched control antibodies.

### *In Vitro* DC–T cell assays

DCs were magnetically enriched from the spleen of naïve mice of each of the four mouse strains (WT, CD40^−/−^, MyD88^−/−^, and DKO) as described above. 1.5 × 10^5^ DCs were incubated with OVA protein, OVA_323–339_ peptide (Sigma Aldrich), or heat-killed *Salmonella* expressing OVA (HKS_OVA_). HKS_OVA_ was prepared by incubating *Salmonella*-OVA (1 × 10^10^ bacteria/mL in 1 mL of PBS) at 65°C for 30 min. The volume was then adjusted with RPMI-1640 to a final bacterial concentration of 1.5 × 10^8^ colony-forming units (CFU)/mL and aliquots were stored at −20°C. DCs were pulsed with HKS_OVA_ at a ratio of four bacteria per DC for 2 h and the cells were washed three times with RPMI-1640 containing 25 μg/mL gentamicin. For the data in Figure 4, 6 × 10^5^ OT-II cells were added and after 1, 6, 24, or 48 h the cells were washed in ice-cold PBS, lysed in RNA lysis buffer, and stored at −20°C until further analysis. For the data in Figure 3, CellTrace Violet (CTV; Life Technologies)-labeled CD4^+^ T cells were added to the HKS_OVA_-pulsed DCs. After 5 days, supernatants were removed and stored at −20°C until assayed for IFN-γ or IL-10. Cells were then harvested, stained with anti-TCR, Ly5.1, and CD4, as well as a cocktail of anti-NK1.1, CD11c, CD11b, and CD19 conjugated to the same fluorochrome to use in an exclusion channel, and analyzed by flow cytometry.

For experiments using OVA protein or OVA_323–339_ peptide, LPS was depleted using Detoxi-Gel endotoxin removing columns (Pierce Biotechnology, Rockford, IL, USA) following the manufacturer’s instructions. LPS was depleted to 1.1 endotoxin unit/mg of protein. 1.5 × 10^5^ DCs enriched as above were incubated with 2 mg/mL OVA protein or 111 ng/mL OVA_323–339_ peptide for 2 h. Cells were washed and CFSE-labeled Ly5.1^+^ CD4^+^ OT-II cells were added as above. After 5 days, supernatants were collected and cells were harvested and stained as above.

### Quantitative real-time PCR

Cells were lysed and homogenized using a QIAshredder (QIAGEN) according to the manufacturer’s instructions. Total RNA was extracted with the High Pure RNA Tissue Kit (Roche Life Science) according to the manufacturer’s protocol. RNA quantity and purity was measured using a NanoDrop ND-1000 Spectrophotometer (NanoDrop Technologies, Inc., Wilmington, DE, USA). RNA was reverse transcribed with the Transcriptor First Strand cDNA Synthesis Kit (Roche) and qPCR was performed on a LightCycler480 (Roche) using LightCycler480 SYBR Green Master (Roche). Primers for hypoxanthine-guanine phosphoribosyltransferase (HPRT) (fwd-TCC TCC TCA GAC CGC TTT T; rws-CCT GGT TCA TCA TCG CTA ATC), TICAM-2 (fwd-GAA GAT CGA AGA GCC TCG TG; rws-GTG ATT GAG ACG CCT TAG CC), NIK (fwd-CTG CAA CCT GAC GGC CTA; rws-CTC CGT GCC AGG AAT GTA GT), β-catenin (fwd-GCA GCA GCA GTT TGT GGA; rws-TGT GGA GAG CTC CAG TAC ACC), A20 (fwd-TCA TCG AAT ACA GAG AAA ATA AGC AG; rws-AGG CAC GGG ACA TTG TTC T), ABIN-2 (fwd-GAC GCA CTT CTG GAT CAG GT; rws-CGC TCC GTA AGT CTT TCA ACT T); and cRel (fwd-TTG CAG AGA TGG ATA CTA TGA AGC; rws-CAC CGA ATA CCC AAA TTT TGA A) were designed using the Universal Probe Library Design platform (Roche) and purchased from Eurofins MWG Operon (Ebersberg, Germany). Specificity and efficiency was tested in initial analyses. Differential gene expression was assessed using the 2^ΔΔCt^-method (24) normalizing to the Ct-value of HPRT and gene expression in stimulated T cells without DCs was used as the reference group.

### Cytokines

IFN-γ was measured using the IFN-γ ELISA set (BD OptEIA, BD Bioscience) and IL-10 was detected using the IL-10 ELISA set (LEGEND MAX, BioLegend) following the manufacturer’s recommendation.

### Statistical analysis

**Figure 1:**
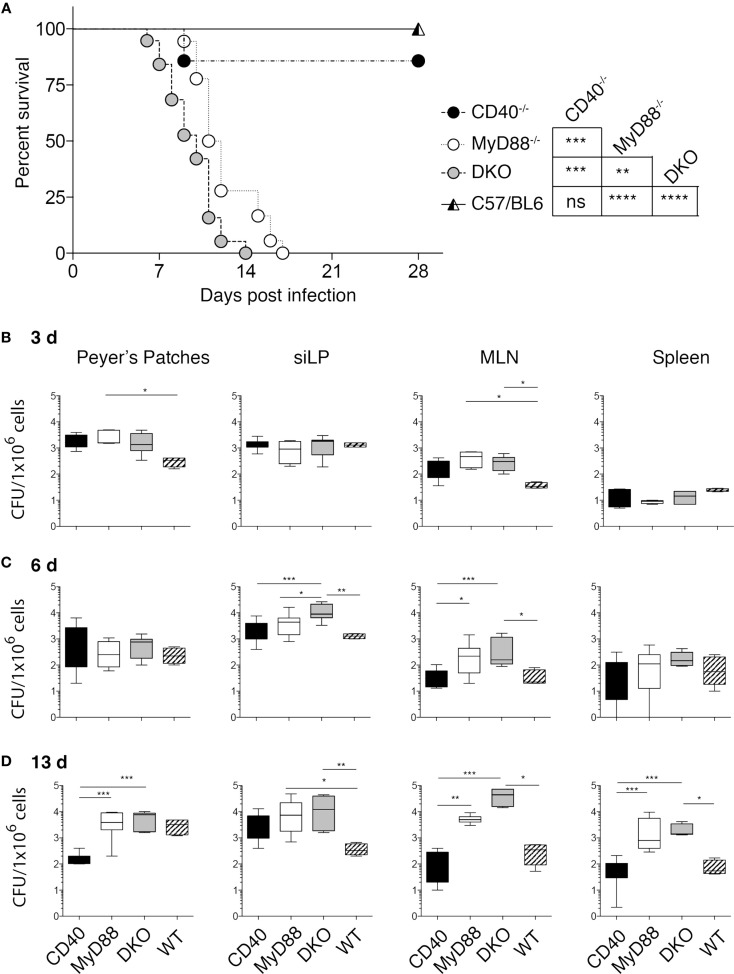
**DKO mice are highly susceptible to Salmonella infection**. **(A)** CD40^−/−^, MyD88^−/−^, DKO, and WT mice were orally infected with 2 × 10^8^ χ4550-OVA and survival was monitored for 2 weeks. Survival of mice is depicted in a Kaplan–Meier plot. Statistical significance between survival curves was assessed using the Mantel–Cox test and results are indicated to the right. Data are pooled from four experiments with a total of 18 CD40^−/−^, 19 MyD88^−/−^, 22 DKO, and 23 WT mice. The drop in survival of CD40^−/−^ reflects the death of one mouse. **(B–D)** *Salmonella* CFUs in PP, siLP, MLN, and spleen are shown for 3 **(B)**, 6 **(C)**, and 13 **(D)** days pi. Data are pooled from two to four independent experiments with a total of 24 CD40^−/−^, 24 MyD88^−/−^, 24 DKO and 18 WT mice. Statistical differences were determined using a Mantel–Cox test **(A)** and Kruskal–Wallis test followed by Dunn’s multiple comparison test **(B–D)**. Error bars indicate SD. Statistical significance is indicated by asterisks as defined in the materials and methods. All other comparisons are non-significant.

Statistical analyses were performed with GraphPad Prism 6.0 (GraphPad Software, La Jolla, CA, USA). The Mantel–Cox test was used for statistical analysis in the survival curve (Figure 1A). For comparison of two independent groups, the two-tailed non-parametric Mann–Whitney-*U* test was applied. Kruskal–Wallis test followed by Dunn’s multiple comparison test or two-way ANOVA followed by Tukey’s multiple comparison test was used for comparison between three or more groups. *p*-values <0.05 were considered significant. *p < 0.05; **p < 0.01; ***p < 0.001; ****p < 0.0001.

## Results

### MyD88^−/−^ and DKO mice are compromised in survival to *Salmonella* infection, but show intact myeloid cell recruitment and DC activation

Studies using purified agonists, such as TLR ligands and agonistic anti-CD40 antibodies have revealed a cooperative influence of CD40 co-stimulation and MyD88-dependent signaling on DC function and the immune response to protein antigen (15, 16, 20, 21). However, whether these pathways act cooperatively to influence the immune response to a bacterial pathogen, which naturally expresses multiple TLR ligands, is unknown. We thus generated mice deficient in both CD40 and MyD88 and orally infected them with *S. typhimurium* χ4550-OVA. Infected DKO mice succumbed earlier than mice lacking MyD88 alone, while WT and CD40^−/−^ mice survived the infection without signs of severe disease (Figure 1A).

To determine the ability of the different mouse strains to control bacterial replication, CFUs in gut-associated tissues (PP, siLP, and MLN), as well as in the spleen, were quantitated at different times post-infection (pi). No significant differences in CFUs between DKO, MyD88^−/−^, and CD40^−/−^ mice at day 3 pi were found (Figure 1B). In PP at day 3 pi, CFUs of MyD88^−/−^ mice were higher than those of WT mice, while in MLN the CFUs of both MyD88^−/−^ and DKO were higher than those of WT mice. By contrast, CFUs in spleen were low in all infected groups (Figure 1B). At day 6 pi, CFUs in siLP and MLN of DKO mice were significantly higher than those in WT and CD40^−/−^ mice, while CFUs in PP and spleen were similar in all groups (Figure 1C). Consistent with the survival data, DKO and MyD88^−/−^ mice had significantly higher CFUs relative to CD40^−/−^ and WT mice at day 13 pi (Figure 1D).

Increased CFUs in DKO and MyD88^−/−^ mice early after infection could be due to differences in recruitment of innate cells with anti-bacterial function, such as neutrophils and inflammatory monocytes (25–28). However, no significant differences in these populations were apparent at day 6 or 13 pi (Figure S1 in Supplementary Material). Thus, bacterial control and host survival are strongly influenced by MyD88 deficiency, while CD40 deficiency influences susceptibility to *S. typhimurium* χ4550 only in combination with MyD88 deficiency. Moreover, the observed survival differences are independent of defects in recruitment of neutrophils and inflammatory monocytes.

As differences in survival to *S. typhimurium* infection between WT and CD40^−/−^ mice relative to DKO and MyD88^−/−^ mice became apparent 7–10 days pi, we hypothesized that an impaired adaptive immune response toward *Salmonella* may be underlying this observation. We first analyzed the DC compartment in gut-associated tissues for the abundance of CD103^+^CD11b^−^, CD103^+^CD11b^+^, and CD103^−^CD11b^+^ DCs, which can differentially impact the T cell response (29). No significant difference in the frequency of these DC subsets was found in the different mouse strains infected with *Salmonella* (Figure S2 in Supplementary Material). Likewise, no difference in the frequency of CD103^+^CD11b^−^ or CD103^−^CD11b^+^ DCs in the spleen of infected mice was apparent except for slightly reduced CD103^+^CD11b^−^ DCs at day 6 pi (Figure S3B in Supplementary Material). However, we observed a relative paucity of CD103^+^CD11b^+^ (P2) DCs in the siLP of CD40^−/−^ mice at day 6 pi (Figure S2C in Supplementary Material, middle panel) although not statistically significant. This could reflect migration and/or death of the cells at day 6 pi. We next investigated the expression of CD80 and CD86 on DC subsets and found no significant difference on the DC subsets in spleen (Figures S3C,D in Supplementary Material) or gut-associated tissues (data not shown). Moreover, the frequency of CD4^+^ and CD8^+^ T cells was not significantly different between the mouse strains in the organs analyzed at day 6 or 13 pi (data not shown). Thus, the observed differences in survival of the mouse strains to oral *Salmonella* infection are independent of differential recruitment of myeloid cells, DC abundance, and expression of CD80 and CD86 on DCs in infected tissues.

### Infected MyD88^−/−^ and DKO mice lack IFN-γ and show elevated systemic IL-10

**Figure 2:**
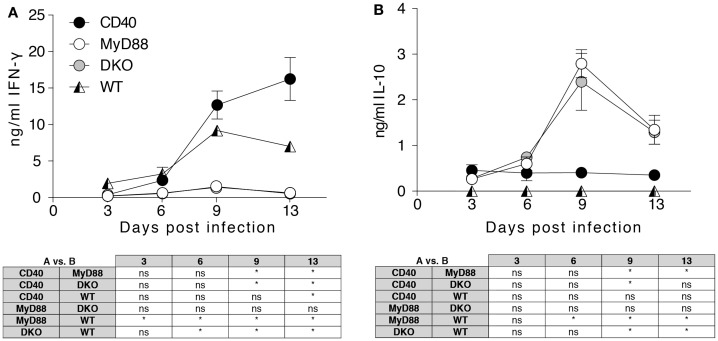
**MyD88-dependent signaling is necessary to initiate a systemic Th1 response to *Salmonella***. Serum samples were collected from the indicated mice at days 3, 6, 9, and 13 pi and screened for cytokines. Results for IFN-γ **(A)** and IL-10 **(B)** are depicted. Results are pooled from two independent experiments with a total of 4–6 mice per group and time point. Statistical significance at indicated time points between groups was assessed using the two-way ANOVA followed by Tukey’s multiple comparison test. Results are depicted in the tables below the graphs. Statistical significance is indicated by asterisks. ns, non-significant.

IFN-γ is critical in controlling *Salmonella* infection (30, 31), and differential production of this cytokine during infection of the different mouse strains could impact survival. Initial experiments used multiplex analysis to assess Th1/Th2/Th17/Th22 cytokines and significant differences were found for IFN-γ and IL-10, which were verified by ELISA. While IFN-γ was significantly increased in the serum of infected WT and CD40^−/−^ mice, MyD88^−/−^ and DKO mice lacked detectable IFN-γ (Figure 2A). By contrast, infected MyD88^−/−^ and DKO mice showed increased serum IL-10 that peaked at day 9 pi while no IL-10 was detected in infected WT or CD40^−/−^ mice (Figure 2B). Thus, during *S. typhimurium* infection, WT and CD40^−/−^ mice produce the Th1 cytokine IFN-γ, while MyD88^−/−^ and DKO mice produce IL-10 but not IFN-γ.

### DCs lacking both MyD88 and CD40 induce increased IL-10 to bacterial antigen and reduced T cell proliferation to protein antigen

**Figure 3:**
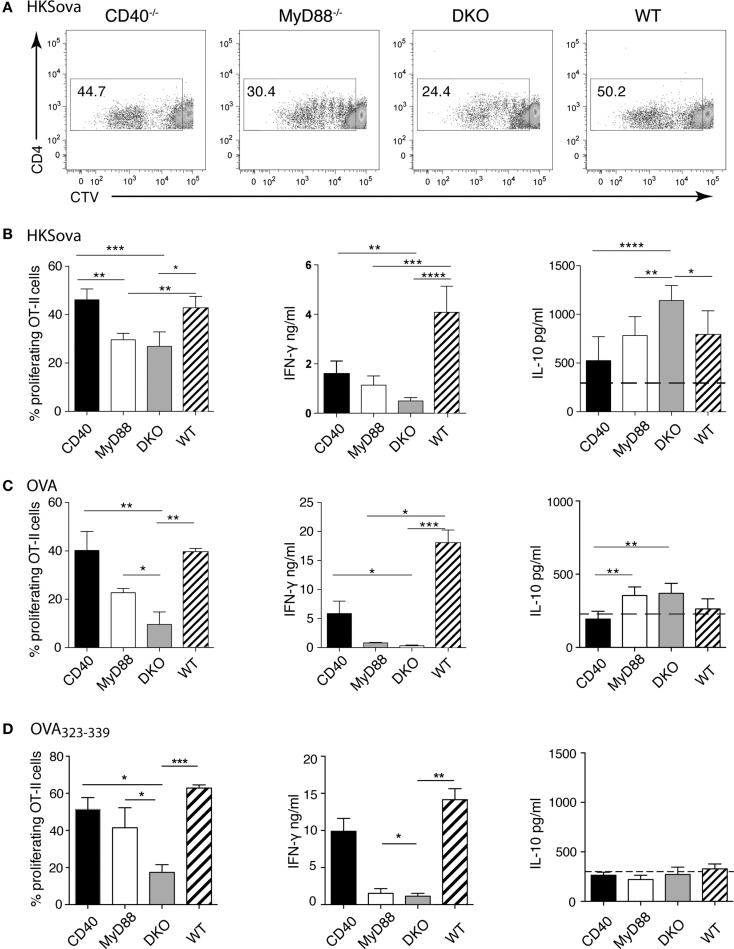
**MyD88-dependent TLR signaling is indispensable to initiate efficient T cell proliferation and Th1 differentiation**. *Ex vivo* T cell proliferation assays were performed using DCs isolated from the spleen of the indicated mice pulsed with HKS_OVA_ **(A,B)**, OVA protein **(C)**, or OVA_323–339_ peptide **(D)** followed by addition of CTV-labeled OT-II cells for 5 days. **(A)** Representative dot plots showing CTV dilution as a measurement of cell division after 5 days of co-incubation with HKS_OVA_-pulsed DCs. **(B–D)** The frequency of proliferating OT-II cells, as well as IFN-γ and IL-10 measured in the same co-culture supernatants, stimulated by DCs from the indicated mouse strain pulsed with **(B)** HKS_OVA_, **(C)** OVA protein, and **(D)** OVA_323–339_ peptide (111 ng) are shown. Black bars represent DCs from CD40^−/−^, white bars from MyD88^−/−^, gray bars from DKO, and hatched bars from WT mice. Horizontal dashed lines in the right panels indicate the detection limit for IL-10. Results are pooled from two **(A,B)**, three **(C)**, or two to three **(D)** independent experiments with a total of 7 **(A,B)** or 9 **(C,D)** mice/group. Error bars indicate SD and statistical significance between groups was assessed using Kruskal–Wallis test followed by Dunn’s multiple comparison test. Statistical significance is indicated by asterisks. All other comparisons are non-significant.

The data thus far suggest that differences in DC subset abundance or CD80/86 expression on DCs, as well as CD4^+^ or CD8^+^ T cell numbers in infected tissues, do not explain the observed difference in survival of the mouse strains to *Salmonella* infection. Instead, differences in serum levels of IFN-γ versus IL-10 may contribute to survival differences. At later times during infection, predominant sources of these cytokines are T cells rather than innate immune cells. We thus examined the ability of isolated splenic DCs to drive proliferation and cytokine production of antigen-specific CD4^+^ T cells. To this end, DCs were pulsed with HKS_OVA_ prior to co-incubation with OVA-specific OT-II T cells for 5 days. DCs from both CD40^−/−^ and WT animals induced significantly higher OT-II proliferation and IFN-γ production than DCs from MyD88^−/−^ or DKO mice (Figures 3A,B). No difference in T cell proliferation or in IFN-γ production between HKS_OVA_-pulsed MyD88^−/−^ and DKO DCs was detected (Figures 3A,B). However, supernatants from co-cultures containing HKS_OVA_-pulsed DKO DCs contained significantly more IL-10 compared to DCs from all other mouse strains (Figure 3B).

Previous studies examining CD40-TLR cooperation in DC activation were performed using purified protein or peptide antigen (15, 20, 32, 33). We thus performed co-culture experiments using DCs pulsed with LPS-depleted OVA protein or OVA_323–339_ peptide. Similar to the results using HKS_OVA_ as antigen, WT and CD40^−/−^ DCs pulsed with OVA protein were significantly better in inducing T cell proliferation (Figure 3C, left) and IFN-γ production (Figure 3C, middle) than MyD88^−/−^ or DKO DCs. DKO mice were significantly less efficient than MyD88^−/−^ mice in inducing OT-II proliferation to both OVA protein and OVA_323–339_ peptide, while this was not the case for HKS_OVA_-pulsed DCs (Figures 3B–D, left). Moreover, with OVA protein as antigen, DCs from WT mice produced more IFN-γ relative to OVA-pulsed DCs from the other genotypes (Figure 3C, middle). Little or no difference in IFN-γ production was apparent between MyD88^−/−^ and DKO DCs pulsed with any of the three antigens (Figures 3B–D, middle).

Interestingly, we could not detect IL-10 production above baseline when MyD88 sufficient DCs were stimulated with OVA protein (Figure 3C, right). By contrast, IL-10 in co-culture supernatants of MyD88^−/−^ or DKO DCs pulsed with OVA protein was significantly higher than IL-10 in supernatants of CD40^−/−^ DCs (Figure 3C, right). Moreover, using HKS_OVA_-pulsed DCs, IL-10 production in co-cultures with DKO DCs was higher than DCs from all other mouse strains (Figure 3B, right). By contrast, OVA_323–339_ peptide-pulsed MyD88-deficient DCs did not elicit IL-10 above background (Figure 3D, right). In general, DCs pulsed with HKS_OVA_ produced the greatest amount of IL-10 relative to OVA or OVA_323–339_ peptide. Since CD40 has been described as an essential survival factor for DCs *in vivo* (6), we assessed if lack of CD40 influenced survival of DCs in the co-cultures. However, parallel cultures stained with Annexin V to assess viability revealed no significant differences in survival of DCs from MyD88^−/−^ or DKO mice relative to each other or to WT or CD40^−/−^ DCs (data not shown).

Thus, combined deficiency of MyD88 and CD40 in DCs adversely affects OT-II proliferation to OVA protein and OVA_323–339_ peptide, but not HKS_OVA_, relative to MyD88 deficiency alone. By contrast, DCs lacking MyD88 alone or both MyD88 and CD40, pulsed with any of the three antigens tested, have a similar ability to induce IFN-γ which is lower than that of DCs from WT or CD40^−/−^ mice. Moreover, DKO DCs pulsed with HKS_OVA_, but not with protein or peptide antigen, show increased IL-10 in culture supernatants relative to DCs lacking only MyD88.

### MyD88-dependent signaling is necessary for gene regulation

**Figure 4:**
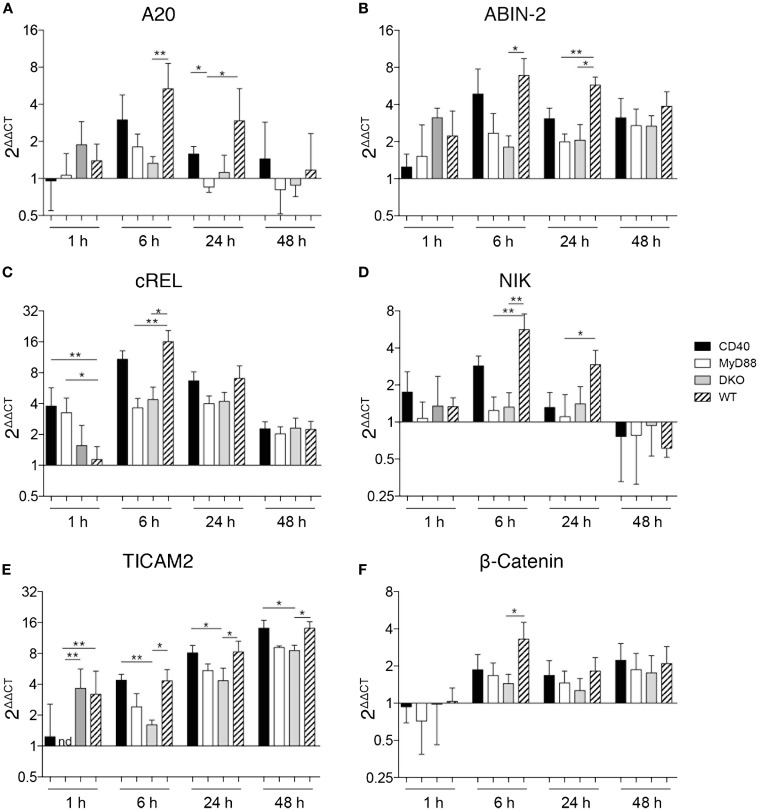
**MyD88-dependent signaling is necessary for gene regulation**. DCs were isolated from the spleen of the indicated mice and stimulated with HKS_OVA_ prior to co-incubation with OT-II cells. Co-incubation was stopped after 1, 6, 24, or 48 h and gene expression was analyzed by qPCR. Gene expression is depicted as fold change against control reactions without HKS_OVA_ normalized against HPRT expression (2^ΔΔCT^). Gene expression of A20 **(A)**, ABIN-2 **(B)**, cRel **(C)**, NIK **(D)**, TICAM-2 **(E)**, and β-catenin **(F)** at the indicated time points are depicted. Black bars represent DCs from CD40^−/−^, white bars from MyD88^−/−^, gray bars from DKO, and hatched bars from WT mice. Results are pooled from two to three independent experiments with a total of 7–12 mice per group and time point assessed in duplicates. Bars depict the mean ± SD. Statistical significance between groups was assessed using Kruskal–Wallis test followed by Dunn’s multiple comparison test. Statistical significance is indicated by asterisks. All other comparisons are non-significant.

Bidirectional crosstalk between DCs and antigen-specific T cells results in induction of signaling pathways in both cell types that influence their cytokine production. We thus assessed the kinetics of gene expression in DCs co-cultured with T cells. Initial experiments included methods to separate DCs and T cells after the co-culture using different washing steps or CD4^+^ MACS beads to magnetically separate CD4 T cells from DCs. This resulted in a loss of cells and flow cytometry analyses revealed that up to 40% of CD4^+^ T cells remained in the DC fraction even after separation (data not shown). This may be due to the tight interaction between DCs and T cell following co-culture. In the absence of an effective way to separate DCs and T cells after co-culture without significant cell loss, we used RNA isolated from HKS_ova_-stimulated OT-II T cells without DCs as the reference against which differential gene expression was normalized. While WT and CD40^−/−^ DCs pulsed with HKS_OVA_ and co-cultured with OT-II cells resulted in differential expression of NFκB- and NFκB2-dependent genes, alterations in gene expression were relatively small in co-cultures containing DCs from MyD88^−/−^ and DKO mice (Figure 4). A20 and ABIN-2, inhibitors of NFκB- and NFκB2 activation, respectively, were initially upregulated in WT and CD40^−/−^ DCs before returning to basal expression levels 48 h post co-incubation (Figures 4A,B). Expression of these genes was higher 6 h post co-incubation in WT DCs compared to the other DCs, particularly DKO DCs (Figures 4A,B). A similar pattern was observed for cRel and NIK, activators of NFκB and NFκB2, respectively (Figures 4C,D). Early expression of cRel in DCs from CD40^−/−^ and MyD88^−/−^ mice co-cultured for 1 h with OT-II cells, however, was higher than in DKO and WT DCs (Figure 4C). The MyD88-independent adaptor molecule for TLR4 signaling, TICAM-2, was increased in co-cultures with DCs from all four mouse strains over time with a higher upregulation in WT and CD40^−/−^ DCs than in MyD88^−/−^ and DKO DCs (Figure 4E). By contrast, the NFκB-independent gene β-catenin was only significantly upregulated in WT DCs 6 h post co-incubation (Figure 4F). Thus, HKS_OVA_-pulsed DCs from WT and CD40^−/−^ mice have higher induction of NFκB-dependent and -independent genes upon co-culture with OT-II cells than MyD88^−/−^ and DKO DCs. Moreover, combined deficiency of MyD88 and CD40 did not alter expression of any of the genes examined relative to MyD88 deficiency alone upon stimulation with HKS_OVA_.

## Discussion

Using purified antigens and agonistic ligands, it has been demonstrated that MyD88-dependent signaling can act in synergy with CD40 stimulation to fully license DCs and induce competent Th1 cells (15, 20). By generating mice deficient in both MyD88 and CD40, we set out to elucidate whether synergy between MyD88 and CD40 influences the anti-bacterial immune response during infection with a live pathogen. We showed that MyD88 deficiency is predominant *in vivo* and overshadows an additional influence of CD40 signaling. This is exemplified by the highly susceptible yet similar response of both MyD88^−/−^ and DKO mice to oral *Salmonella* infection and similar CFUs in infected tissues.

Even though DKO mice succumbed to *Salmonella* infection earlier than MyD88^−/−^ mice, both strains had low serum IFN-γ and elevated IL-10. Thus, in the absence of MyD88, IL-10-producing T cells instead of Th1 cells predominate in infected mice as reflected in serum cytokine levels. It has been demonstrated that, in the absence of Th1 cells, infected mice produce large quantities of IL-10 and readily succumb to infection with attenuated *aroA*^−^*aroD*^−^ *S. typhimurium* (34). The increase in serum IL-10 may indicate functional skewing toward Treg differentiation, which has been linked to inadequate CD40 signaling (17, 35) and the strength and duration of T cell activation (7). Indeed, our co-culture experiments showed that DCs lacking both MyD88 and CD40 had more IL-10 in the supernatant relative to DCs lacking MyD88 alone with HKS_OVA_ as stimulus. This differed from *in vivo* infection data where infected MyD88^−/−^ and DKO mice showed similar levels of elevated serum IL-10. This may be attributed to the global nature of the genetic deletions in the knockout mice. That is, an influence of all cells lacking the genes is apparent *in vivo* while only DCs lacked the gene(s) in the co-culture system. Indeed, TLRs and CD40 on T cells can also influence effector functions (36, 37); the lack of these signals in T cells *in vivo* may thus also differentially influence results from *in vivo* experiments versus defined co-culture systems. *In vivo*, *Salmonella*-infected mice lacking CD40 had somewhat higher IFN-γ than WT mice, which was not apparent in *in vitro* co-cultures for likely the same reasoning regarding *in vitro* versus *in vivo* settings. The somewhat higher IFN-γ in the serum of *Salmonella*-infected CD40^−/−^ mice differs from the requirement for CD40 in IFN-γ production by *Leishmania*-infected Balb/c mice (17) and IFN-γ-producing T cells at early, but not later, time points in a graft versus host disease model (35). Different experimental models, the genetic background of the mouse strain, and time points examined may factor into the requirement of CD40 for production of different cytokines *in vivo*.

To reflect the *in vivo* infections, initial co-culture experiments used HKS_OVA_ as antigen that, as discussed above, revealed an influence of CD40 beyond that of MyD88 with respect to IL-10 production. However, DCs lacking MyD88 alone or both MyD88 and CD40 similarly drove proliferation of, and IFN-γ by, OT-II cells in co-culture experiments using HKS_OVA_ as antigen. To determine how the complexity of the antigen influenced a synergistic effect of MyD88 and CD40 on DC function, co-cultures were also performed using OVA protein or OVA_323–339_ peptide. Interestingly, both of these purified antigens revealed a synergistic effect of MyD88 and CD40 on OT-II proliferation with little if any effect on IFN-γ production. As we did not add an agonistic anti-CD40 antibody to our co-cultures, these results are consistent with previous findings (20, 38). Overall, data from the co-culture experiments using the three types of antigen (HKS_OVA_, OVA, and OVA_323–339_ peptide) showed that synergistic effects of MyD88 and CD40 may be apparent on some (IL-10 production) but not all (induction of proliferation and IFN-γ production) DC functions, depending on the complexity of the antigen. Indeed, synergistic effects observed using purified, well-defined antigens may not necessarily be revealed when complex antigens, such as bacteria, challenge the immune system where the diverse array of antigens and receptor ligands displayed by bacteria trigger numerous pathways in multiple cell types to facilitate host survival.

Consistent with the proliferation and IFN-γ results from co-culture experiments, a synergistic effect of MyD88 and CD40 on induction of NFκB-dependent and -independent genes in DCs was not apparent using bacteria as antigen. That is, MyD88 deficiency was predominant and a synergistic effect of CD40 and MyD88 on gene expression was not apparent. This supports that the activation of DCs via MyD88-dependent signaling is predominant during the activation of T cells. Upregulation of TICAM-2, an additional TLR4-binding adapter for MyD88-independent DC activation (39), may contribute to MyD88-independent activation of DCs in response to HKS_OVA_.

Overall, our data using mice with a single or double deficiency in MyD88 and CD40 revealed that synergistic effects of CD40 and MyD88 do not influence host survival to *Salmonella* infection, CFUs in infected tissues or serum levels of IFN-γ or IL-10. However, synergistic effects of CD40 and MyD88 become apparent when analyzing DC-driven aspects of T cell activation in co-cultures, such as proliferation and cytokine production, and depend on the complexity of the antigen used. Our data contrast previous studies showing that signaling via MyD88 and CD40 together is needed to initiate IL12p70 secretion and Th1 differentiation (15, 20). However, these *in vivo* studies were carried out using LPS-free purified protein antigen and agonistic CD40 antibodies. Instead, we used the complex, multi-ligand live pathogen *Salmonella*, which allowed analysis of the mechanisms that take place during bacterial infection. Overall, our data support that the complexity of the antigen analyzed can differentially influence the ability of DCs to drive CD4 T cell proliferation and cytokine production *in vitro*. Moreover, the effects of DC function on T cells observable in defined cultures *in vitro* may not translate into differences in host survival to infection with bacterial pathogens where multiple cell types and complex cytokine environments determine the ultimate outcome to bacterial challenge.

## Conflict of Interest Statement

The authors declare that the research was conducted in the absence of any commercial or financial relationships that could be construed as a potential conflict of interest.

## Supplementary Material

The Supplementary Material for this article can be found online at http://journal.frontiersin.org/article/10.3389/fimmu.2015.00460

## References

[1] Takeuchi O Akira S. Pattern recognition receptors and inflammation. Cell (2010) 140:805–20. 10.1016/j.cell.2010.01.022 20303872

[2] Kawasaki T Kawai T. Toll-like receptor signaling pathways. Front Immunol (2014) 5:461. 10.3389/fimmu.2014.00461 25309543 PMC4174766

[3] Gray CM Remouchamps C McCorkell KA Solt LA Dejardin E Orange JS Noncanonical NF-κB signaling is limited by classical NF-κB activity. Sci Signal (2014) 7:ra13. 10.1126/scisignal.2004557 24497610 PMC3960999

[4] Sun S-C. The noncanonical NF-κB pathway. Immunol Rev (2012) 246:125–40. 10.1111/j.1600-065X.2011.01088.x 22435551 PMC3313452

[5] Kawai T Akira S. Toll-like receptors and their crosstalk with other innate receptors in infection and immunity. Immunity (2011) 34:637–50. 10.1016/j.immuni.2011.05.006 21616434

[6] Bennett SR Carbone FR Karamalis F Flavell RA Miller JF Heath WR. Help for cytotoxic-T-cell responses is mediated by CD40 signalling. Nature (1998) 393:478–80. 10.1038/30996 9624004

[7] Macagno A Napolitani G Lanzavecchia A Sallusto F. Duration, combination and timing: the signal integration model of dendritic cell activation. Trends Immunol (2007) 28:227–33. 10.1016/j.it.2007.03.008 17403614

[8] Lanzavecchia A Sallusto F. Progressive differentiation and selection of the fittest in the immune response. Nat Rev Immunol (2002) 2:982–7. 10.1038/nri959 12461571

[9] Luft T Maraskovsky E Schnurr M Knebel K Kirsch M Görner M Tuning the volume of the immune response: strength and persistence of stimulation determine migration and cytokine secretion of dendritic cells. Blood (2004) 104:1066–74. 10.1182/blood-2003-12-4146 15113760

[10] Watts TH. TNF/TNFR family members in costimulation of T cell responses. Annu Rev Immunol (2005) 23:23–68. 10.1146/annurev.immunol.23.021704.115839 15771565

[11] Xiao G Harhaj EW Sun SC NF-κB-inducing kinase regulates the processing of NF-κB2 p100. Mol Cell (2001) 7:401–9. 10.1016/S1097-2765(01)00187-3 11239468

[12] Lind EF Ahonen CL Wasiuk A Kosaka Y Becher B Bennett KA Dendritic cells require the NF-κB2 pathway for cross-presentation of soluble antigens. J Immunol (2008) 181:354–63. 10.4049/jimmunol.181.1.354 18566401

[13] Warger T Osterloh P Rechtsteiner G Fassbender M Heib V Schmid B Synergistic activation of dendritic cells by combined toll-like receptor ligation induces superior CTL responses in vivo. Blood (2006) 108:544–50. 10.1182/blood-2005-10-4015 16537810

[14] Krummen M Balkow S Shen L Heinz S Loquai C Probst H-C Release of IL-12 by dendritic cells activated by TLR ligation is dependent on MyD88 signaling, whereas TRIF signaling is indispensable for TLR synergy. J Leukoc Biol (2010) 88:189–99. 10.1189/jlb.0408228 20360404

[15] Edwards AD Manickasingham SP Spörri R Diebold SS Schulz O Sher A Microbial recognition via toll-like receptor-dependent and -independent pathways determines the cytokine response of murine dendritic cell subsets to CD40 triggering. J Immunol (2002) 169:3652–60. 10.4049/jimmunol.169.7.3652 12244157

[16] Fujii S-I Shimizu K Smith C Bonifaz L Steinman RM. Activation of natural killer T cells by α-galactosylceramide rapidly induces the full maturation of dendritic cells in vivo and thereby acts as an adjuvant for combined CD4 and CD8 T cell immunity to a coadministered protein. J Exp Med (2003) 198:267–79. 10.1084/jem.20030324 12874260 PMC2194082

[17] Martin S Agarwal R Murugaiyan G Saha B. CD40 expression levels modulate regulatory T cells in *Leishmania donovani* infection. J Immunol (2010) 185:551–9. 10.4049/jimmunol.0902206 20525887

[18] Ballesteros-Tato A León B Lee BO Lund FE Randall TD. Epitope-specific regulation of memory programming by differential duration of antigen presentation to influenza-specific CD8^+^ T cells. Immunity (2014) 41:127–40. 10.1016/j.immuni.2014.06.007 25035957 PMC4233138

[19] Seah SGK Brady JL Carrington EM Ng WC Sutherland RM Hancock MS Influenza-induced, helper-independent CD8^+^ T cell responses use CD40 costimulation at the late phase of the primary response. J Leukoc Biol (2013) 93:145–54. 10.1189/jlb.0612266 23108101

[20] Schulz O Edwards AD Schito M Aliberti J Manickasingham S Sher A CD40 triggering of heterodimeric IL-12 p70 production by dendritic cells in vivo requires a microbial priming signal. Immunity (2000) 13:453–62. 10.1016/S1074-7613(00)00045-5 11070164

[21] Fujii S-I Liu K Smith C Bonito AJ Steinman RM. The linkage of innate to adaptive immunity via maturing dendritic cells in vivo requires CD40 ligation in addition to antigen presentation and CD80/86 costimulation. J Exp Med (2004) 199:1607–18. 10.1084/jem.20040317 15197224 PMC2212806

[22] Sundquist M Wick MJ. TNF-α-dependent and -independent maturation of dendritic cells and recruited CD11c^int^CD11b^+^ cells during oral *Salmonella* infection. J Immunol (2005) 175:3287–98. 10.4049/jimmunol.175.5.3287 16116221

[23] Rydström A Wick MJ. Monocyte recruitment, activation, and function in the gut-associated lymphoid tissue during oral *Salmonella* infection. J Immunol (2007) 178:5789–801. 10.4049/jimmunol.178.9.5789 17442963

[24] Pfaffl MW Relative quantification. In: Dorak MT, editor. Real-time PCR. Taylor & Francis Group: Garland Science (2006). p. 63–82.

[25] Tam MA Rydström A Sundquist M Wick MJ. Early cellular responses to *Salmonella* infection: dendritic cells, monocytes, and more. Immunol Rev (2008) 225:140–62. 10.1111/j.1600-065X.2008.00679.x 18837781

[26] Rydström A Wick MJ. Monocyte and neutrophil recruitment during oral *Salmonella* infection is driven by MyD88-derived chemokines. Eur J Immunol (2009) 39:3019–30. 10.1002/eji.200939483 19839009

[27] Rydström A Wick MJ. *Salmonella* inhibits monocyte differentiation into CD11c^hi^ MHC-II^hi^ cells in a MyD88-dependent fashion. J Leukoc Biol (2010) 87:823–32. 10.1189/jlb.0909615 20124491

[28] Hawley KL Olson CM Iglesias-Pedraz JM Navasa N Cervantes JL Caimano MJ CD14 cooperates with complement receptor 3 to mediate MyD88-independent phagocytosis of *Borrelia burgdorferi*. Proc Natl Acad Sci U S A (2012) 109:1228–32. 10.1073/pnas.1112078109 22232682 PMC3268315

[29] Bekiaris V Persson EK Agace WW. Intestinal dendritic cells in the regulation of mucosal immunity. Immunol Rev (2014) 260:86–101. 10.1111/imr.12194 24942684

[30] Mittrücker HW Kaufmann SH. Immune response to infection with *Salmonella typhimurium* in mice. J Leukoc Biol (2000) 67:457–63. 10770276 10.1002/jlb.67.4.457

[31] Bao S Beagley KW France MP Shen J Husband AJ. Interferon-gamma plays a critical role in intestinal immunity against *Salmonella typhimurium* infection. Immunology (2000) 99:464–72. 10.1046/j.1365-2567.2000.00955.x 10712678 PMC2327174

[32] Ahonen CL Doxsee CL McGurran SM Riter TR Wade WF Barth RJ Combined TLR and CD40 triggering induces potent CD8^+^ T cell expansion with variable dependence on type I IFN. J Exp Med (2004) 199:775–84. 10.1084/jem.20031591 15007094 PMC2212721

[33] Reis e Sousa C Hieny S Scharton-Kersten T Jankovic D Charest H Germain RN In vivo microbial stimulation induces rapid CD40 ligand-independent production of interleukin 12 by dendritic cells and their redistribution to T cell areas. J Exp Med (1997) 186:1819–29. 10.1084/jem.186.11.1819 9382881 PMC2199158

[34] Ravindran R Foley J Stoklasek T Glimcher LH McSorley SJ. Expression of T-bet by CD4 T cells is essential for resistance to *Salmonella* infection. J Immunol (2005) 175:4603–10. 10.4049/jimmunol.175.7.4603 16177105

[35] Ferrer IR Wagener ME Song M Kirk AD Larsen CP Ford ML. Antigen-specific induced Foxp3^+^ regulatory T cells are generated following CD40/CD154 blockade. Proc Natl Acad Sci U S A (2011) 108:20701–6. 10.1073/pnas.1105500108 22143783 PMC3251074

[36] Reynolds JM Dong C. Toll-like receptor regulation of effector T lymphocyte function. Trends Immunol (2013) 34:511–9. 10.1016/j.it.2013.06.003 23886621

[37] Martin S Pahari S Sudan R Saha B. CD40 signaling in CD8^+^CD40^+^ T cells turns on contra-T regulatory cell functions. J Immunol (2010) 184:5510–8. 10.4049/jimmunol.0902762 20400702

[38] Baruah P Dumitriu IE Malik TH Cook HT Dyson J Scott D C1q enhances IFN-γ production by antigen-specific T cells via the CD40 costimulatory pathway on dendritic cells. Blood (2009) 113:3485–93. 10.1182/blood-2008-06-164392 19171874

[39] Seya T Oshiumi H Sasai M Akazawa T Matsumoto M. TICAM-1 and TICAM-2: toll-like receptor adapters that participate in induction of type 1 interferons. Int J Biochem Cell Biol (2005) 37:524–9. 10.1016/j.biocel.2004.07.018 15618008

