# Supplementary figures and images for: Synergy between CD40 and MyD88 Does Not Influence Host Survival to Salmonella Infection
Source: Front Immunol. 2015 Sep 14;6:460. doi: 10.3389/fimmu.2015.00460 (PMC4568434; doi:10.3389/fimmu.2015.00460)

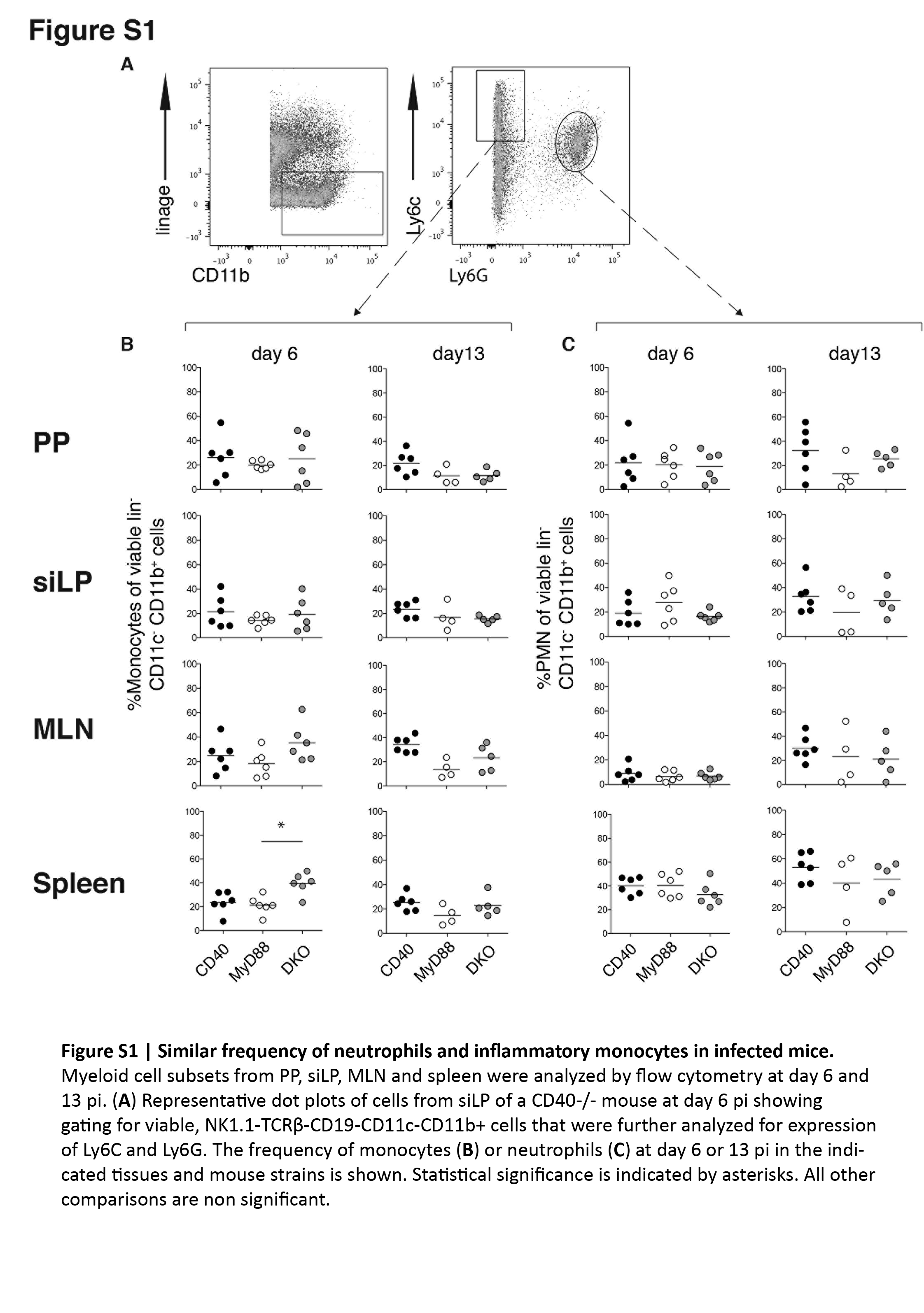

Supplement: Supplementary file 1 [file Image_1.TIF]

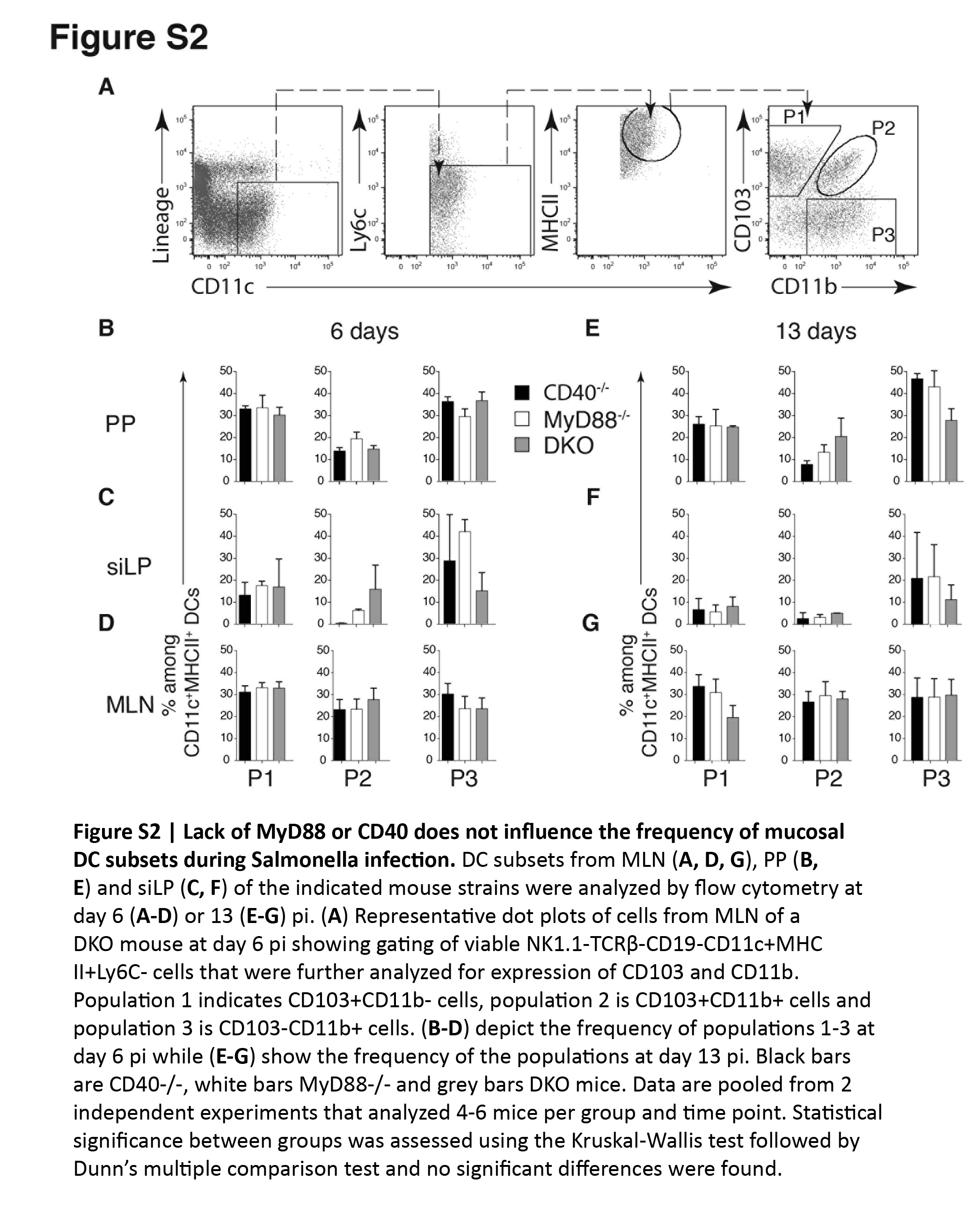

Supplement: Supplementary file 2 [file Image_2.TIF]

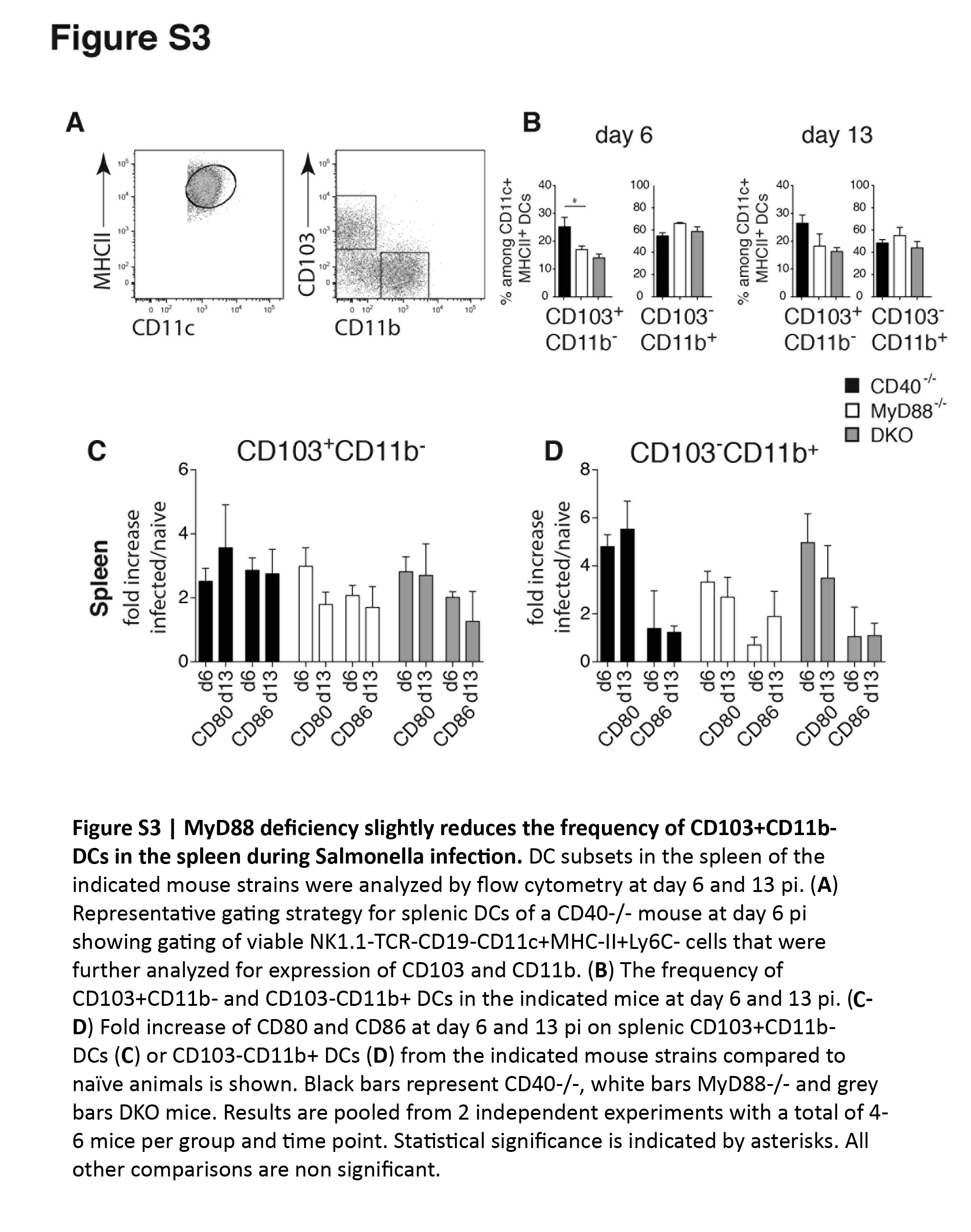

Supplement: Supplementary file 3 [file Image_3.TIF]
